# The Association between Blood Lead Levels and Osteoporosis among Adults—Results from the Third National Health and Nutrition Examination Survey (NHANES III)

**DOI:** 10.1289/ehp.9716

**Published:** 2007-03-12

**Authors:** James R. Campbell, Peggy Auinger

**Affiliations:** Department of Pediatrics, University of Rochester Medical Center, Rochester, New York, USA

**Keywords:** blood lead levels, bone mineral density, osteoporosis

## Abstract

**Background:**

Osteoporosis is a reduction in bone mass sufficient to increase the risk of fracture. Lead exposure during childhood may be a risk factor for low bone mineral density (BMD). Basic-science research demonstrates that lead exposure is associated with a decrease in BMD in animals. However, human studies are limited.

**Objective:**

Our objective was to conduct a secondary analysis of a national database to explore the association between lead exposure and osteoporosis in adult humans.

**Methods:**

In this study we used data from the Third National Health and Nutrition Examination Survey (NHANES III). We analyzed subjects who were ≥ 50 years of age. A concurrent venous blood lead level defined lead exposure. The primary outcome variable was the BMD of the total hip. We conducted analyses on four groups: non-Hispanic white men, non-Hispanic white women, African-American men, and African-American women. We conducted bivariate analyses between covariates known to be associated with bone density (i.e., age, body mass index, calcium intake, ethanol/tobacco consumption, physical activity, socioeconomic status) and the total hip BMD. The significant covariates were introduced into analysis of covariance to determine the association between BMD and blood lead level tercile.

**Results:**

The adjusted mean total hip BMD among non-Hispanic white males with a blood lead level in the lowest tercile versus the highest tercile was 0.961 g/cm^2^ and 0.934 g/cm^2^, respectively (*p* < 0.05). We also found a similar association among white females, but the difference was marginally significant (0.05 < *p* < 0.10).

**Conclusions:**

We found a significant inverse association between lead exposure and BMD, but only among white subjects. However, because of the cross-sectional design of NHANES, we cannot make inferences about the temporal sequence of this association. With the large number of adults who had lead exposure in the past and the morbidity associated with osteoporosis, further inquiry is necessary on the possible casusal association between lead exposure and osteoporosis in humans.

Osteoporosis is a reduction in bone mass sufficient to increase the risk of fractures (Krane and Holick 1998), and is a common and serious affliction of the elderly (Chandler et al. 2000; Looker et al. 1995; Melton et al. 1992). Lead exposure may be a risk factor in the development of osteoporosis. Animal studies report that increased lead exposure is associated with a decrease in bone density (Escribano et al. 1997; Gruber et al. 1997; Puzas et al. 1999) and bone strength (Ronis et al. 2001). Additional *in vitro* studies report that lead exposure inhibits the function of chondrocytes (Hicks et al. 1996) and osteoblasts, the cells that manufacture bone (Klein and Wiren 1993; Puzas et al. 1999; Ronis et al. 2001).

Human studies on this association are limited. In a study of children, Laraque et al. (1990) found no association between bone density and lead exposure. However, because the children were examined at a young age (range, 18–47 months), sufficient time may not have elapsed for the adverse effects on the bone to become manifest. In a cross-sectional study of 1,021 adults age 16–81 years, Alfvén et al. (2002) also found no association between lead exposure and bone mineral density (BMD). However, the mean blood lead level was low among the subjects studied [3.1 μg/dL (Alfvén T, personal communication)]—suggesting that the lack of an association may have been attributed to low exposure. In contrast, an epidemiologic study on whether postmenopausal bone resorption is associated with elevated blood lead levels found an inverse association between blood lead level (geometric mean blood lead level, 2.9 μg/dL among postmenopusal women) and BMD (Nash et al. 2004). In a study of former workers from a lead smelting facility, there was an inverse association between the log of the current blood lead level and spine BMD [regression coefficient −0.039; 95% confidence interval (CI), −0.060 to −0.019; *p* < 0.0002] (Potula et al. 2006).

As recently as the late 1970s, 78% of the U.S. population had blood lead levels ≥ 10 μg/dL (Mahaffey et al. 1982), the current threshold of concern defined previously by the Centers for Disease Control (CDC 1991). Bone is a repository for 90–95% of the total body burden of lead (Wedeen 1992), and harbors it for years after initial exposure (Gulson et al. 1995). Thus, a high proportion of adult Americans may currently have elevated bone lead levels. With this many who were exposed to lead when younger, and the morbidity associated with osteoporosis, it is important to investigate whether an association exists between lead exposure and osteoporosis in humans. Our objective was to conduct a secondary analysis of a national database to explore the association between lead exposure and osteoporosis in a large number of adults.

## Methods

### NHANES III survey

We used data from the Third National Health and Nutrition Examination Survey (NHANES III), conducted by the National Center for Health Statistics (NCHS) of the CDC (NCHS 1998). NHANES III is a national-probability, cross-sectional survey of noninstitutionalized Americans. It uses multistage, nonrandom sampling techniques, and thus results were weighted to be representative of the U.S. population. Personal household interviews and physical examinations of about 40,000 people ≥ 2 months of age were conducted between 1988 and 1994 (NCHS 1994). Participants gave written informed consent before entering the study, with parents consenting for children.

The Household Adult Questionnaire was administered to subjects ≥ 17 years of age, and asked an extensive series of questions on their health (NCHS 1994). This included questions on the presence and location of back pain, and past history, location, and mechanism of fractures (NCHS 1994). The answers to these questions were used to define clinical-based outcome variables. Questions regarding covariates (e.g., medications, medical conditions, calcium intake, tobacco use, ethanol use, physical activity) were also asked. Blood lead levels were assayed on all subjects ≥ 1 year of age. BMD measurements were conducted on all men and nonpregnant women ≥ 20 years of age who underwent the physical examination.

### Study sample

In the present investigation we focused on the 8,654 subjects ≥ 50 years of age who took part in the Household Adult Questionnaire. We defined this cutoff because it is the age group in which primary osteoporosis occurs. From this group, subjects were excluded if they did not have a blood lead level and/or a BMD measurement (*n* = 2,430) or if they had an ethnicity other than non-Hispanic African American or non-Hispanic white (*n* = 1,420). Because few women were premenopausal (*n* = 115), we restricted the analysis to postmenopausal women. The remaining 4,689 subjects made up the primary sample, which underwent analysis.

### Definition of lead exposure

We defined lead exposure as the blood lead level at the time the subject was interviewed by the NHANES survey. Blood was collected by venous phlebotomy and stored in EDTA-anticoagulant tubes that were prescreened for background lead contamination. We performed duplicate lead analyses on each sample, and recorded the arithmetic mean of the duplicates for each participant. Blood lead was assayed by graphite-furnace atomic absorption spectrophotometry (Gunter et al. 1996). Blood lead levels below the detection limit were assigned a value of 0.7 μg/dL.

### Definition of BMD and clinical outcomes

The primary outcome was the mean BMD of the total hip. BMD measurements were obtained using dual-energy X-ray absorptiometry (DEXA) instruments (QDR 1000; Hologic, Waltham, MA) in pencil-beam mode. A quality control program was used to ensure data quality (Wahner et al. 1994). The sites examined were limited to the hip: the femoral neck, trochanter, intertrochanter, Ward’s triangle, and total hip (NCHS 1994). We limited our analyses to the total hip because the BMD of the hip subregions are highly correlated with the BMD of the total hip (*r* = 0.76–0.98) (Kalkwarf et al. 2003).

Two clinical outcomes were defined: the presence of back pain, and whether the subject had an osteoporotic-related fracture. The vertebrae are the most common bone to be fractured due to osteoporosis, often resulting in back pain (Krane and Holick 1998). We categorized subjects as having back pain if they responded on the Household Adult Questionnaire as having had lower and/or middle back pain over the preceding 12 months. The second clinical measure of osteoporosis was based on whether the subject had a fracture due to osteoporosis. Subjects were classified as having had a fracture due to osteoporosis if they had a hip, wrist, or vertebral fracture [sites most commonly affected by osteoporosis (Krane and Holick 1998)] at ≥ 50 years of age that occurred because of a fall from a standing height or less. Subjects were classified as not having had a fracture due to osteoporosis if they reported never having had a fracture, having had a fracture at < 50 years of age, or having had a fracture at ≥ 50 years of age caused by a fall farther than from a standing height, from a car accident or other severe trauma.

### Definition of covariates

In adjusted analysis, we considered several confounders: age, race, sex, body mass index (BMI), menopausal status, tobacco use, alcohol use, physical activity, calcium intake, chronic medical conditions, use of certain medications, and socioeconomic status.

Race and sex are strongly associated with bone density (Cooper et al. 1992; Nelson et al. 1997; Snelling et al. 2001; Turner et al. 1998). We therefore conducted analyses stratified by sex and race (i.e., non-Hispanic white men, non-Hispanic white women, African-American men, and African-American women).

Higher weight is associated with a decreased risk of osteoporosis (CDC 1998; Seeman et al. 1983; Snelling et al. 2001). As a measure of body mass, we used the BMI, defined as the weight in kilograms divided by the square of the height in meters.

Menopausal status is strongly associated with bone density (Krane and Holick 1998; Nash et al. 2004). Because the NHANES database does not provide a single variable defining a woman’s menopausal status, we created a variable to categorize women as premenopausal or postmenopausal. The following criteria (Kalkwarf et al. 2003) were applied sequentially so that the successive rules were applied only to women not already categorized: *a*) age ≥ 60 years, postmenopausal; *b*) bilateral oophorectomy, postmenopausal; *c*) a period or pregnancy within the previous 12 months, pre-menopausal; *d*) follicle-stimulating hormone level > 40 IU/L, postmenopausal; *e*) current use of oral contraceptive pill, premenopausal; *f* ) age 50–59 years, postmenopausal. We found that few women were premenopausal (*n* = 115). We thus restricted the analysis to postmenopausal women; therefore, menopausal status was not adjusted in the analysis.

Smoking tobacco is associated with an increased risk of osteoporosis (Seeman et al. 1983). Subjects were classified into those who never smoked, former smokers, and current smokers.

Alcohol consumption is associated with an increased risk of osteoporosis (Seeman et al. 1983). Subjects reported the average number of alcoholic beverages consumed per day and the number of days that an alcoholic beverage was consumed over the previous 12 months. These two terms were multiplied together and divided by 12 to give a measure of the average number of drinks consumed per month over the previous year.

Increased physical activity is associated with a decreased risk of hip fractures (Cooper et al. 1988; Lau et al. 1988; Snelling et al. 2001). Subjects were asked about the frequency at which they conducted 13 physical activities (i.e., walking, jogging, bicycling, swimming, doing aerobics, dancing, doing calisthenics, gardening, lifting weights, and four “other” categories) over the previous month. A measure of monthly physical activity was defined by summing the number of times that each of 13 physical activities was performed. Although this is a measure of current physical activity, studies report that current physical activity is correlated to physical activity over a person’s lifetime (Hirvensalo et al. 2000; Rhodes et al. 1999).

Increased calcium intake is associated with increased bone density (Johnston et al. 1992; Turner et al. 1998). Subjects were asked about past intake of milk. Subjects specified the frequency of milk consumption (never, less than once per week, once per week, less than once per day but more than once per week, once per day, more than once per day) for each of several age strata (i.e., 5–12, 13–17, 18–35, 36–65, and > 65 years). As a proxy of calcium intake, we calculated a measure of milk consumption during bone anabolism—from childhood until roughly the mid-thirties. We assigned each frequency of milk consumed during an age stratum (never, less than once per week, once per week, less than once per day but more than once per week, once per day, more than once per day) an ordinal value (respectively, 0–5). We calculated the proportion of years an age stratum made up the 5- to 35-year age span (for example, the 5- to 12-year age strata makes up 0.26 of the 5- to 35-year age span). We multiplied the ordinal value representing the frequency of milk consumption during an age stratum by the proportion of years the corresponding age stratum made up the 5- to 35-year age span. Three age strata were used (i.e., 5–12 years, 13–17 years, 18–35 years). Each of the three resulting terms were added to achieve a weighted estimate of weekly milk consumption between 5 and 35 years of age.

Medications may also affect bone density. We identified whether the subject reported taking medications that adversely affect bone density (i.e., anticonvulsants, calcitonin, cyclosporine, cytotoxic drugs, estrogens, gluco-corticoids, progesterones). This outcome was defined dichotomously (i.e., currently using or not using medications that affect bone density). We also defined a second measure based on whether the female subjects had even been on any kind of estrogen replacement therapy.

The presence of certain chronic medical conditions—such as thyroid disease—is known to cause changes in bone density. We identified whether a physician had ever told the subject that she or he had thyroid disease. This outcome was defined dichotomously. Other chronic medical conditions (i.e., arthritis, heart failure, stroke, bronchitis, emphysema, gout, and cancers) were not controlled using this measure because they would affect BMD via altered levels of physical activity and/or use of medications. These latter two variables are already included in the regression model.

We used educational level as a proxy of socioeconomic status. Other measures of socioeconomic status, such as poverty index ratio, had many missing values and were nevertheless moderately correlated to educational level (Pearson correlation = 0.47; *p* < 0.001).

### Analyses

We analyzed the association between current blood lead level and total hip BMD among four groups: non-Hispanic white men, non-Hispanic white women, African-American men, and African-American women. To identify covariates for adjusted analysis, we conducted bivariate analysis (for each aforementioned age- and sex-defined group) between the variables described above and the total hip BMD. For continuous covariates, we calculated Pearson correlation coefficients and the corresponding two-tailed *p*-value. For categorical covariates, we used the *t*-test to compare mean BMD and calculated the corresponding two-tailed *p*-value. Covariates found to be sizable (i.e., *p* ≤ 0.20) were analyzed in a correlation matrix to assess for collinearity; we found that within each sex- and race-defined group, no covariates were multicollinear (i.e., correlation coefficient > 0.50) with each other. We decided *a priori* to introduce age and BMI as covariates into all adjusted analyses for each of the four sex- and race-defined groups. In addition, we identified the following additional covariates as significant: for non-Hispanic white males, education level; for African-American males, education level, presence of chronic medical condition, and calcium intake; for non-Hispanic white females, education level and use of estrogen replacement; for African-American females, education level, use of estrogen replacement, and calcium intake.

The significant covariates were introduced into analysis of covariance (ANCOVA) to determine the independent association between BMD of the total hip and blood lead level tercile. We assessed each model for assumptions required by ANCOVA (Leech et al. 2005). We found, for each age- and sex-defined group, that BMD was normally distributed within each blood lead level tercile; further, there was homogeneity of variance of the BMD between each blood lead level tercile. To achieve the assumption of homogeneity of regression slopes of the continuous covariates for each blood lead level tercile, we log-transformed BMI in the analysis of the non-Hispanic white males; no transformations were required for the remaining age-and sex-defined groups. Finally, to account for the assumption that observations must be independent, we used SUDAAN software to adjust for the multistage, nonrandom sampling design of the survey (Shah et al. 1991).

In the unadjusted analysis of the clinical outcomes, for each of the four sex- and race-defined groups, we calculated the proportion of subjects with lower back pain and with fractures due to osteoporosis as a function of blood lead level tercile. We noted that the differences in BMD between blood lead level terciles diminished in adjusted analyses. Because of this, and because the comparisons of the proportions of the unadjusted clinical outcomes were small and not significant, we did not conduct adjusted analysis of the clinical outcomes.

## Results

The demographic data of the four race- and sex-defined groups are presented in Table 1. The adjusted analyses revealed a significant inverse association between lead exposure and BMD among the non-Hispanic white male subjects (Table 2). The adjusted mean total hip BMD among non-Hispanic white males with a blood lead level in the lowest versus the highest tercile was 0.961 gm/cm^2^ versus 0.934 gm/cm^2^ (*p* < 0.05). An association with marginal significance was discerned among non-Hispanic white females. The adjusted mean total hip BMD among non-Hispanic white females with a blood lead level in the lowest versus the highest tercile was 0.789 gm/cm^2^ versus 0.771 gm/cm^2^ (*p* = 0.09). No association was discerned among African-American subjects. In addition, we found that none of the comparisons of the clinical outcomes (i.e., prevalence of back pain, osteoporotic fractures) by blood lead level tercile were significant.

## Discussion

Our study found a significant inverse association between lead exposure and BMD, but only among white subjects (Table 2). Although there was a negative trend between blood lead level tercile and BMD among the African-American subjects, the differences were not significant. This lack of significance may be attributed to a considerably smaller sample size of the African-American subjects than the white subjects.

No association was found between blood lead level tercile and clinical outcomes. A possible explanation may be the relatively small difference in BMD between blood lead tercile. Clinical trials of bisphosphanates that found differences in clinical outcomes such as fracture rates reported corresponding differences in BMD of ≥ 5% (Black et al. 1996; Bone et al. 1997; Liberman et al. 1995). Among the non-Hispanic white subjects in our study, the difference in BMD between the low tercile and high tercile was about 2–3%. This difference in BMD may be too small to discern a significant difference in clinical outcomes. Another possible reason for a lack of an association is that neither clinical outcome may be valid as a measure of osteoporotic fractures. Not all osteoporotic vertebral fractures cause pain. Conversely, it is possible other causes of back pain obscured the pain due to osteoportic vertebral fractures.

It is possible that our findings of an inverse association between lead exposure and BMD is spurious because of inadequately controlled covariates. For example, the measure of calcium intake required a subject to recall the quantity of milk consumed during their youth. However, if their recall was not reliable, the use of this measure in adjusted analysis could lead to inadequate control of a potential confounder. In another example, we controlled for the use of current medications. However, because current medication use may not reliably measure effects of past medication use, its use in adjusted analysis could again lead to inadequate control of a potential confounder.

A limitation of this study is the cross-sectional design of the NHANES database. Although we found a significant inverse association, it is unclear whether past lead exposure caused a lower BMD, or whether an elevated blood lead level is a result of a lower BMD. In other words, it is possible that increased bone turnover produced an increase in blood lead from the endogenous release of lead from the bone. Using stable lead isotope fingerprinting methods, Gulson et al. (1998, 2002) found that lead can be mobilized from the skeleton into the blood. This issue has been raised in the literature. Nash et al. (2004), using NHANES III, also found an inverse association between BMD and blood lead level among perimenopausal and postmenopausal women, and concluded that lead stored in bone may significantly increase blood lead levels. However, because of the cross-sectional design of NHANES, they acknowledged not being able make inferences about the temporal sequence of this association. The same is certainly true of our study.

Despite the previous limitation, it is possible the association found in this study reflects lead as causative in the development of osteoporosis. *In vitro* studies show that lead adversely affects growth plate chondrocytes by altering cell phenotype and function (Hicks et al. 1996). Other *in vitro* research reports that lead inhibits osteoblast phenotype and function (Bonucci 1981; Klein and Wiren 1993; Puzas et al. 1992). The strongest evidence in support of causation comes from randomized trials on animals. Histomorphometry of the growth plates of lead-exposed rats shows defective remodeling, altered growth plate thickness due to the loss of proliferating cells, and disorganization of the growth plate architecture (Hamilton and O’Flaherty 1994). Escribano et al. (1997) found that histomorphometric measures of bone density (i.e., bone volume, trabecular number, and trabecular thickness) were lower in lead-exposed rats than controls. Gruber et al. (1997) found that lead-exposed rats (mean blood lead level 21 μg/dL) had lower DEXA-based BMD than controls. Other researchers have also shown that lead exposure decreases BMD or bone strength in rats (Puzas et al. 1999; Ronis et al. 2001), and inhibits fracture healing in mice (Carmouche et al. 2005).

As described in the introduction, a high proportion of adult Americans may currently have elevated bone lead levels. With this large number of lead-exposed adults, and the morbidity associated with osteoporosis, it is important to investigate further whether a causative association exists between lead exposure and osteoporosis in humans.

## Figures and Tables

**Table 1:** Characteristics of the race- and sex-specified groups.

	Female		Male	
Characteristic	White (*n* = 1,754)	African-American (*n* = 629)	White (*n* = 1,693)	African-American (*n* = 613)
Mean age (years)	66.7	64.3	64.1	62.9
Mean BMI [kg/m^2^ (range)]	27.2 (11.7–52.2)	29.5 (13.3–58.5)	27.2 (15.9–48.4)	26.8 (15.0–48.8)
Education level (%)				
Less than high school	31.6	53.6	32.7	57.9
High school	40.2	26.2	28.0	23.5
More than high school	27.8	19.4	38.9	17.7
Missing	0.4	0.8	0.4	0.9
Monthly alcohol use (%)				
Nondrinker	67.9	80.2	44.8	51.1
< 30 drinks	21.8	14.7	33.4	26.6
30–120 drinks	8.3	2.3	15.6	12.6
> 120 drinks	0.7	0.0	4.6	3.8
Missing	1.3	2.8	1.6	5.9
Smoking status (%)				
Nonsmoker	55.0	58.0	25.2	25.2
Former smoker	29.2	18.0	54.4	37.9
Current smoker	15.8	24.0	20.4	36.9
Monthly physical activity^a^ (%)				
None	21.5	37.3	11.3	22.5
< 30	46.0	39.5	47.5	45.1
30–60	25.1	19.2	29.0	22.9
> 60	7.4	4.0	12.2	9.5
Weekly milk ingestion^b^ (%)				
Never	3.5	2.0	1.3	2.6
< 1 per week	8.0	14.0	4.9	9.9
1 per week	10.1	10.6	8.4	12.4
< 1 per day, > 1 per week	22.2	26.6	22.1	29.4
1 per day	29.7	24.8	33.4	27.5
> 1 per day	21.8	17.9	27.2	16.1
Missing	4.7	4.1	2.7	2.1
Medication use^c^ (%)				
Present	20.6	9.7	3.3	2.8
Not present	79.4	90.3	96.7	97.2
Mean BLL [μg/dL (range)]	3.6 (0.7–28.7)	4.5 (0.7–23.3)	4.9 (0.7–48.1)	7.7 (0.7–52.9)
Mean total hip BMD [g/cm^2^ (range)]	0.778 (0.300–1.320)	0.880 (0.450–1.450)	0.946 (0.500–1.620)	1.021 (0.540–1.980)

BLL, blood lead level.

a Number of physical activities conducted over the previous month.

b Weekly milk ingestion during ages 5–35 years.

c Medications that adversely affect bone density.

**Table 2:** BMD and clinical measures by group and blood lead level tercile.

	Female		Male	
Measure	White	African-American	White	African-American
Adjusted mean (SE) BMD (g/cm^2^)^a^				
Lowest	0.789 (0.006)	0.898 (0.010)	0.961 (0.007)	1.036 (0.011)
Middle	0.776^b^ (0.006)	0.882 (0.009)	0.944^c^ (0.006)	1.023 (0.010)
Highest	0.771^b^ (0.007)	0.873 (0.012)	0.934^c^ (0.009)	1.011 (0.013)
Unadjusted proportion with back pain (%)^a^				
Lowest	29.5	22.3	30.5	24.8
Middle	28.7	25.4	26.8	20.4
Highest	27.5	23.8	29.8	30.8
Unadjusted proportion with osteoporotic (%) fractures^a,d^				
Lowest	5.7	3.0	1.1	0.2
Middle	6.2	1.0	0.4	0.4
Highest	8.2	1.8	1.0	0.4

a Blood lead level tercile.

b 0.05 < *p* < 0.10 in comparison to lowest tercile.

c *p* < 0.05 in comparison to lowest tercile.

d Fractures of the hip, wrist, and/or vertebrae at ≥ 50 years of age that occurred because of a fall from a standing height or less.
